# The risk of low energy availability in Chinese elite and recreational female aesthetic sports athletes

**DOI:** 10.1186/s12970-020-00344-x

**Published:** 2020-03-04

**Authors:** Kun Meng, Junqiang Qiu, Dan Benardot, Amelia Carr, Longyan Yi, Jieting Wang, Yiheng Liang

**Affiliations:** 1 Arts School, Beijing Sport University, Beijing, China; 2 Department of Exercise Biochemistry, Exercise Science School, Beijing Sport University, Beijing, China; 3 Department of Nutrition, Georgia State University, Atlanta, GA USA; 4 Center for the Study of Human Health, Emory University, Atlanta, GA USA; 5 Centre for Sport Research, Deakin University, Burwood, VIC Australia; 6 Department of Kinesiology and Health Education, University of Texas at Austin, Austin, TX USA

**Keywords:** Aesthetic sports, Female athletes, Low energy availability, Eating disorder, Bone mineral density

## Abstract

**Background:**

Low energy availability (LEA) is a medical condition observed in athletes, with a higher prevalence in aesthetic sports. For the first time, this study evaluated the relative prevalence of LEA in female elite athletes (ELA) and recreational athletes (REA) in aesthetic sports in China.

**Methods:**

Female athletes from 6 sports (trampolining, rhythmic gymnastics, aerobics, dance sport, cheerleading and dance) were recruited, including ELA (*n* = 52; age = 20 ± 3) on Chinese national teams and REA (*n* = 114; Age = 20 ± 2) from Beijing Sport University. Participants completed 2 online questionnaires to assess LEA and eating disorder risk. These included the Low Energy Availability in Females Questionnaire (LEAF-Q), which provided information on injury history, gastrointestinal function and menstrual history, and the Eating Disorder Inventory-3 Referral Form (EDI-3 RF). For a sub-group of elite athletes (*n* = 14), body composition, bone mineral density, and blood serum were also quantified.

**Results:**

A total of 41.6% of participants (*n* = 69) were at increased risk of LEA, and 57.2% of participants (*n* = 95) were classified as high in eating disorder risk. For ELA vs. REA, there was a significantly higher prevalence of LEA risk (55.8% vs. 35.1%; *p* = 0.012) and amenorrhea (53.8% vs. 13.3%; *p* < 0.001). Elite athletes at increased risk of LEA had significantly lower estradiol (*p* = 0.021) and whole-body BMD (*p* = 0.028). Pearson correlations indicated that the whole-body BMD (*r* = − 0.667, *p* = 0.009) correlated negatively with LEAF-Q score.

**Conclusions:**

Results of this study indicate that there is a risk of LEA in female Chinese athletes within aesthetic sports, and significantly higher prevalence of increased LEA risk observed in ELA than in REA. Chinese coaches and sports medicine staff working elite female athletes in aesthetic sports should develop strategies to reduce the prevalence of LEA.

## Introduction

It is essential for athletes to consume sufficient energy, at specific time points (pre, during and post-exercise) to sustain health and performance [1]. Certain groups of athletes are at a higher risk of having energy intakes that fail to fully satisfy the higher physiological requirements of exercise [2]. This failure can result in energy deficiency status (result of insufficient caloric intake and/or excessive energy expenditure [3]) and low energy availability (LEA), which is defined as an individual mismatch between energy intake and exercise, leading to insufficient energy to support normal physiological function [2]. Long-term energy deficiency status can negatively impact athletic performance, menstrual function, bone health, metabolic rate, exercise recovery, immunity, cardiovascular function, and mental health [4, 5].

It has been proposed that LEA may be the driving factor for menstrual disruptions, and may be a major contributing factor to the Female Athlete Triad [6]. In 2007, The American College of Sports Medicine (ACSM) published a position stand on the Female Athlete Triad that identified three interrelated components—LEA with or without disordered eating, menstrual dysfunction, and low bone mineral density [4]. In 2014, the concept of The Female Athlete Triad was expanded to include the multiple performance detriments, and health-related problems experienced by both male and female athletes, in a new model referred to as Relative Energy Deficiency in Sport (RED-S), which has LEA at its core [7]. In the same year, the Low Energy Availability in Females Questionnaire (LEAF-Q) was developed, which is a validated screening tool (78% sensitivity and 90% specificity) for the identification of female endurance athletes at increased risk of LEA [8]. It is recommended that the LEAF-Q is implemented in combination with a validated eating disorders/disordered eating screening tool [9], such as the Eating Disorder Inventory-3 Referral Form (EDI-3 RF) [10]. In addition, measurement of LEA-related biomarkers (e.g., leptin, triiodothyronine (T3), estradiol, testosterone and cortisol) can objectively quantify variables that may contribute toward an individual’s energy deficiency status [11].

Aesthetic sports are defined as those which require well-developed physical capacities (power, speed, endurance, flexibility) as well as technical skill and artistry [12]. In such sports, elite performers typically exhibit a low fat mass, and/or a low body weight, and the scoring has a subjective component. Due to the important role of body shape and appearance in aesthetic sports, athletes often fail to satisfy energy requirements, by limiting energy intake or increasing energy expenditure through excessive exercise training to achieve body composition goals [13], which may elevate the risk of LEA [14].

Chinese elite athletes have previously won Olympic and World Championship medals in aesthetic sports, including trampolining, diving, and rhythmic gymnastics. It has been established that athletes who perform at a high level in aesthetic sports (e.g., rhythmic gymnastics, synchronized swimming, diving, and dancing) in Western countries are at a higher risk of LEA [12, 15–18], however the prevalence of LEA in populations of Chinese athletes has not been established. In China, recreational sports such as Latin dance and aerobics are very popular among women of various ages, and Chinese women participate in such recreational sports in order to maintain physical and mental health, and change their body composition [19]. Severe weight loss programs and unintentional heavy exercise are two factors which may increase the risk of LEA [20], and may within this population. Given the potential for risk of LEA in both elite and recreational Chinese athletes, it is necessary to investigate the incidence of the risk of LEA in aesthetic sports, across these two population groups.

The primary aim of this study was to investigate the risk of LEA in female Chinese athletes in aesthetic sports, for elite athletes (ELA) and recreational athletes (REA) using the LEAF-Q, and to assess the risk of an eating disorder, using Eating Disorder Inventory-3 Referral Form (EDI-3 RF). The secondary aim was to quantify (in 14 ELA), body composition, bone mineral density (BMD), and several serum biofactors (estradiol (E2), triiodothyronine (T3), testosterone, cortisol, leptin and ferritin) to determine their relationship with LEA risk.

## Methods

### Participants

A total of 166 athletes from aesthetic sports, including 52 ELA (age = 20 ± 3 y) from 3 different sports (trampolining, rhythmic gymnastics, aerobics) volunteered to participate in this cross-sectional study. Subjects classified as ELA had recently (within 12 months) competed in a national or international championship event. There were 114 (age = 20 ± 2 y) recreational athletes (REA) from 5 different sports (rhythmic gymnastics, aerobics, dance sport, cheerleading and dance). All REA were currently participating in these sports at Beijing Sport University (BSU). Those classified as REA were not participating at the national/elite level or not classified as an ELA by a sporting organization, but were participating regularly (at least 3 times per week) in one of the selected aesthetic sporting disciplines. Inclusion criteria comprised female participants, aged > 15 years, and regular participation in one of the aesthetic sports, as per the classification criteria stated above for ELA and REA. Participants were excluded from the study if they had a history of chronic illness or metabolic disease. The sub-group of 14 ELA were all current Chinese National Team members, and all were preparing for the 2020 Tokyo Olympic Games. All participants gave signed informed consent in accordance with the latest version of the Declaration of Helsinki and all experimental procedures were approved by the BSU ethics committee (2019109H).

### Questionnaires

#### Low energy availability in females questionnaire (LEAF-Q)

Participants completed the LEAF-Q, which consists of 25 items related to injury history, gastrointestinal function, menstrual function, and the use of contraceptives (e.g., hormonal patches, hormonal ring). There are additional questions on participant demographics, body weight history, previous involvement in sport, training type, and training frequency/volume per week. All completed questionnaires were scored using the LEAF-Q scoring key, with a total score ≥ 8 considered as an increased risk for LEA or Female Athlete Triad. Suggested cut-offs for injuries, gastrointestinal dysfunction and menstrual disturbance are ≥2, ≥2, and ≥ 4, respectively [8]. Primary amenorrhea is defined as the absence of menarche by the age of 15 years. Secondary amenorrhea is defined as the absence of menses for at least 3 months, and oligomenorrhea is defined as menstrual cycles of longer than 35 days in duration [4].

#### Eating disorder Inventory-3 referral form (EDI-3 RF)

Participants completed the validated EDI-3 Referral Form, which is an abbreviated version of the Eating Disorder Inventory-3 (EDI-3) [10]. The EDI-3 RF includes three scales (drive for thinness, bulimia, body dissatisfaction) and five behavioral symptom questions, which are intended to identify individuals with potential eating disorders or eating pathology. Participants were identified as having a risk of eating disorders based on three criteria: the individual’s body mass index (BMI) only; BMI plus responses to EDI-3 questions about excessive eating concerns; and responses to behavioral questions pertaining to eating disorder pathology [10].

#### Body composition and bone mineral density (BMD)

Body composition and bone mineral density were measured using dual-energy X-ray absorptiometry (Lunar iDXA, GE Healthcare, Madison, WI, USA). During the scan, participants remained stationary, in a supine posture, with their arms by their sides and legs together. The typical duration of the whole-body scan was approximately 8–10 minutes (min). BMD was determined for the whole body and for specific body segments (arms, legs, trunk, ribs, hip, and lumbar spine). Low BMD was defined as Z-score between − 1.0 and − 2.0 SD, together with a history of nutritional deficiencies, hypoestrogenism, stress fractures, and/or other secondary clinical risk factors for fracture [4]. DXA-derived body composition values included body mass, fat mass, and lean mass. BMI was calculated as a ratio of weight to height (kg/m^2^).

### Blood samples

Blood samples were obtained after an overnight fast, at 7:30–8.10 am, from an antecubital vein. Samples were collected into a 5 ml gel serum tube (Vacuette, Frickenhausen, Germany) and then analyzed for testosterone, cortisol and ferritin concentration, via an enzyme-labeled analyzer (Beckman DXI 800, Beckman Coulter, Fullerton, CA, USA). Measurement of E2, T3 and leptin conducted using ELISA (Infinite F50, Tecan, Switzerland).

### Statistical analysis

Data were analyzed using SPSS Statistics, version 21. To determine differences between treatment groups (ELA and REA) and EA status (high-risk and low-risk), parametric data were analyzed using an independent sample t-test, and nonparametric data were analyzed using a Mann-Whitney U-Test. Chi-squared tests were used to determine differences in the prevalence of LEA, ED, injury, gastrointestinal dysfunction, and menstrual disturbance between groups. Pearson correlation coefficients were used to determine the presence of any significant relationships. Differences in body composition, BMD and blood measures between subgroups were analyzed using an independent sample t-test. The statistical significance level was set at *p* < 0.05.

## Results

### Risk of LEA in Chinese aesthetic sports

Overall, 29 (55.8%) of ELA and 40 (35.1%) of REA were identified as being at increased risk of LEA (LEAF-Q total score ≥ 8) respectively; there was a significant difference between the two groups. (55.8% vs. 35.1%, χ^2^ = 6.289, *p* = 0.012) (Table 2). For ELA, years of training (mean ± SD) was 11.1 ± 3.7, which was significantly higher than the REA, who had 7.2 ± 3.3 years of training (*p* < 0.001). Similarly, the frequency and total hours per week for training was significantly higher in ELA compared with REA (*p* < 0.001 and *p* < 0.001 respectively) (Table 1). The prevalence of menstrual disturbance (67.3% vs. 43.9%, χ^2^ = 7.858, *p* = 0.005) and primary amenorrhea (53.8% in ELA vs. 13.2% in REA, χ^2^ = 30.802, *p* < 0.001) among ELA were all significantly higher than REA (Table 2).

**Table 1:** Participant characteristics

	Elite athletes (*n* = 52)	Recreational athletes (*n* = 114)	*P*-value
Age (y)	20 ± 3	20 ± 2	0.901
Height (cm)	165.1 ± 6.1	167.3 ± 4.0	0.021*
Weight (kg)	50.7 ± 5.9	53.9 ± 5.3	0.001*
BMI (kg/m^2^)	18.6 ± 1.9	19.3 ± 1.7	0.022*
Years of training (y)	11.1 ± 3.7	7.2 ± 3.3	< 0.001**
Frequency per week (d)	5.3 ± 1.4	3.9 ± 1.4	< 0.001**
Exercise per week (h)	28 (12–43)	8 (6–12)	< 0.001**
LEAF-Q score	8 (5–10)	9 (8–11)	0.031*

Parametric data were summarized as mean ± SD, and nonparametric data were reported by median and interquartile range (25th percentile and 75th percentile)

*LEAF-Q* Low Energy Availability in Females Questionnaire

^*∗*^*P-value < 0.05.*^*∗∗*^*P-value < 0.01*

**Table 2:** The prevalence of physiological symptoms between elite athletes and recreational athletes

	Elite athletes (*n* = 52)	Recreational athletes (*n* = 114)	χ^2^	*P*-value
	%(n)	%(n)		
LEA risk	55.8 (29)	35.1 (40)	6.289	0.012*
ED risk	51.9 (27)	59.6 (68)	0.871	0.351
Underweight	44.2 (23)	30.7 (35)	2.875	0.090
Injury	30.8 (16)	23.7 (27)	0.934	0.334
Gastrointestinal dysfunction	50.0 (26)	59.6 (68)	1.354	0.245
Menstrual disturbance	67.3 (35)	43.9 (50)	7.858	0.005**
Primary amenorrhea	53.8 (28)	13.2 (15)	30.802	< 0.001**
Secondary amenorrhea	30.8 (16)	24.6 (28)	.706	0.401
Oligomenorrhea	13.5 (7)	13.2 (15)	.003	0.957

*LEA* low energy availability, *ED* eating disorder

^*∗*^*P-value < 0.05.*^*∗∗*^*P-value < 0.01*

### Risk of LEA, body composition, BMD, and hormone concentrations in 14 elite athletes

For the sub-group of 14 ELA, eight (57%) were classified as identified as being at increased risk of LEA. There was a significant difference in the LEAF-Q scores between the high-risk athletes (LEAF-Q total score ≥ 8) and the low-risk athletes (LEAF-Q total score < 8) within this sub-group (*p* < 0.001) (Table 3). Athletes who were at risk of LEA had a higher incidence of amenorrhea and lower E2 levels compared with athletes who were not at risk of LEA (*p* < 0.05, *p* = 0.021 respectively) (Tables 4 and 5). However, concentrations for testosterone, cortisol, T3, leptin and ferritin were not significantly different between the two groups (*p* = 0.419, *p* = 0.735, *p* = 0.771, *p* = 0.348, *p* = 0.426 respectively). Compared with the low-risk group, the high- risk group had significantly lower BMD in the arms (*p* = 0.038), legs (*p* = 0.007) and whole-body (*p* = 0.028). There was also a significant difference in total-body BMD Z-score (*p* = 0.015) between two groups. However, no significant difference in body composition (fat mass and lean mass) was identified between the high-risk group and low-risk group (*p* = 0.945, *p* = 0.892 respectively) (Table 6). Pearson correlations indicated that the leg (*r* = − 0.774, *p* = 0.001), trunk (*r* = − 0.551, *p* = 0.041), and whole-body BMD (*r* = − 0.667, *p* = 0.009) correlated negatively with LEAF-Q score. However, no significant correlations were found for any blood indicators related to LEAF-Q score. The whole-body BMD had no significant correlation with E2 (*r* = 0.120, *p* = 0.683). Additionally, training volume correlated negatively with BMI (*r* = − 0.739, *p* = 0.003), ferritin (*r* = − 0.537, *p* = 0.048) and positively with cortisol (*r* = 0.814, *p* < 0.001).

**Table 3:** Summary characteristics divided by classification of risk of low energy availability in 14 elite athletes

	At risk of LEA (*n* = 8)	Not at risk of LEA (*n* = 6)	*P*-value
Age (y)	19 ± 3	21 ± 4	0.313
Height (cm)	164.6 ± 6.1	161.2 ± 7.4	0.358
Weight (kg)	49.6 ± 3.1	49.5 ± 2.1	0.963
BMI (kg/m^2^)	18.0 ± 1.1	19.1 ± 1.4	0.119
Years of training (y)	12.3 ± 2.9	12.9 ± 4.6	0.903
Frequency per week (d)	6.0 ± 0.0	5.8 ± 0.5	0.264
Exercise per week (h)	37.5 ± 14.5	33.0 ± 15.7	0.589
LEAF-Q score	10.3 ± 1.8	4.2 ± 2.1	< 0.001**

Values are presented as means ± SD

*LEAF-Q* Low Energy Availability in Females Questionnaire

^*∗*^*P-value < 0.05.*^*∗∗*^*P-value < 0.01*

**Table 4:** The prevalence of physiological symptoms divided by classification of risk of low energy availability in 14 elite athletes

	At risk of LEA (*n* = 8) %(n)	Not at risk of LEA (*n* = 6) %(n)	χ^2^	*P*-value
ED risk	75.0 (6)	33.3 (2)	2.431	0.119
Underweight	62.5 (5)	33.3 (2)	1.167	0.280
Injury	62.5 (5)	16.7 (1)	2.941	0.086
Gastrointestinal dysfunction	62.5 (5)	16.7 (1)	2.941	0.086
Menstrual disturbance	75.0 (6)	33.3 (2)	2.431	0.119
Primary amenorrhea	87.5 (7)	33.3 (2)	4.381	0.036*
Secondary amenorrhea	50.0 (4)	0.00 (0)	4.200	0.040*
Oligomenorrhea	37.5 (3)	16.7 (1)	0.729	0.393

*ED* eating disorder

^*∗*^*P-value < 0.05.*^*∗∗*^*P-value < 0.01*

**Table 5:** Metabolic and reproductive hormone concentrations in 14 elite athletes and divided by the risk of LEA

	At risk of LEA (*n* = 8)	Not at risk of LEA (*n* = 6)	*P*-value
Testosterone (nmol/L)	1.66 ± 0.25	1.87 ± .066	0.419
cortisol (nmol/L)	418.15 ± 109.31	422.11 ± 109.30	0.735
E2 (pmol/L)	55.49 ± 8.66	67.20 ± 7.34	0.021*
T3 (ng/ml)	73.28 ± 12.81	75.48 ± 14.77	0.771
Leptin (μg/L)	7.45 ± 1.25	6.79 ± 1.25	0.348
Ferritin (μg/L)	45.05 ± 29.22	32.58 ± 26.29	0.426

Values are presented as means ± SD

*E2* estradiol, *T3* triiodothyronine

^*∗*^*P-value < 0.05.*^*∗∗*^*P -value < 0.01*

**Table 6:** Body composition and BMD in 14 elite athletes and divided by the risk of LEA

	At risk of LEA (*n* = 8)	Not at risk of LEA (*n* = 6)	*P*-value
**Body composition**			
Body fat (%)	19.41 ± 2.27	19.47 ± 2.68	0.968
Body fat (kg)	9.61 ± 1.56	9.66 ± 1.64	0.945
Lean mass (%)	75.97 ± 2.14	75.88 ± 2.59	0.943
Lean mass (kg)	37.66 ± 2.79	37.50 ± 1.01	0.892
**Body mineral density**			
BMC (g)	2285.33 ± 234.60	2316.78 ± 201.08	0.797
Arms BMD (g/cm^2^)	0.64 ± 0.04	0.69 ± 0.05	0.038*
Legs BMD (g/cm^2^)	1.13 ± 0.05	1.24 ± 0.08	0.007**
Trunk BMD (g/cm^2^)	0.97 ± 0.05	1.03 ± 0.07	0.095
Rib BMD (g/cm^2^)	0.82 ± 0.06	0.88 ± 0.05	0.062
Hip BMD (g/cm^2^)	1.03 ± 0.07	1.08 ± 0.12	0.284
Lumbar spine BMD (g/cm^2^)	1.09 ± 0.07	1.15 ± 0.10	0.227
Total-body BMD (g/cm^2^)	1.06 ± 0.04	1.14 ± 0.09	0.028*
Total-body BMD Z-score	0.43 ± 0.38	1.08 ± 0.49	0.015*

Values are presented as means ± SD

*BMI* body mass index, *BMC* bone mineral content, *BMD* bone mineral density

^*∗*^*P-value < 0.05.*^*∗∗*^*P-value < 0.01*

## Discussion

### Risk of LEA in Chinese aesthetic sports female athletes

This is the first study to estimate the prevalence of LEA risk in female aesthetic sport athletes in China. In addition, we analyzed biological indicators of LEA in 14 elite athletes, all of whom were national or world-class athletes who were training for the Tokyo 2020 summer Olympic Games. The key findings of the current investigation were that Chinese elite athletes had a higher prevalence of an increased risk of LEA than Chinese recreational athletes (*p* = 0.012), and the elite athletes who were at increased risk of LEA had significantly lower estradiol (*p* = 0.021) and whole-body BMD (*p* = 0.028).

Our findings indicated that there may be a higher prevalence of LEA in female Chinese elite athletes compared with Chinese recreational athletes; 55.8% of elite athletes were at risk of increased LEA, compared to 35.1% of recreational athletes, a difference that was significantly different (*p* = 0.012). Similarly to the results of the current investigation Logue et al. [21] found that the prevalence of the LEA risk as identified via the LEAF-Q, in Irish international and provincial/inter-county athletes, was 1.7 and 1.8-fold more prevalent, respectively, than in recreational athletes. Further, a study investigating elite Australian athletes within 11 Olympic sports [22] reported a 53% prevalence of increased LEA risk as indicated by LEAF-Q results. In contrast, within a population of elite cross-country skiers in Sweden [23] an increased LEA risk was reported in 31% of athletes, again using the same questionnaire (LEAF-Q). Further research is required to investigate the prevalence of LEA within different populations of elite athletes, and may focus on the potential influence of factors including the training and nutritional practices undertaken by athletes of different nationalities.

In the current investigation, 51.9% of elite athletes and 59.6% of recreational athletes were classified as being at risk of ED (using the EDI-3 RF), and 27.1% of the all participants (elite and recreational athletes combined) were identified with being at risk of LEA, coupled with increased eating disorder risk. This percentage of participants at risk of ED is ~ 10% lower than the 69% reported by Robbeson et al. [24] in female dancers in South Africa, using the same questionnaire (EDI-3 RF). Other previous studies have reported the prevalence of eating pathology or eating disorders to be 89.2%, for sports emphasizing leanness in Malaysia [25], and 83% for ballet dancers [26] in the United States. The lower risk of eating disorders and associated risk factors detected in the current investigation, compared with previous findings may be related to some of the typical characteristics of Chinese dietetic culture. It has been reported that 94% of the Chinese population routinely consume three meals per day, and that foods typically consumed at a high frequency (e.g., rice and steamed bread) are high in carbohydrate [27]. In contrast, it has been established that the diets of those identified as having a high risk of ED or LEA are characterized by long-term, intentional restriction of carbohydrate intake [28, 29].

In the current investigation, elite athletes, compared with recreational athletes, had a higher training frequency (mean: 5.3 vs. 3.9 days/week, *p* < 0.001), and higher median training volumes (28 vs. 8 h/week, *p* < 0.001). The significant differences between the two groups may provide some explanation for the increased LEA risk in the elite athletes within the current investigation, in that elevated exercise energy expenditure induced by higher training volumes may contribute to LEA, unless energy intake is increased [2]. It has also been suggested that the relatively high training hours observed for some elite athletes may reduce eating opportunities, contributing further to higher LEA risk [20].

Findings of the current investigation indicated that 53.8% of Chinese elite athletes in aesthetic sports had primary amenorrhea, and that long-term heavy training was reported for elite athletes within the study. A previous study reported that athletes who had engaged in intensive training and participated in competitive sports during childhood may suffer a delay in menarche, especially those in sports that emphasize lean body physique [30]. In the current investigation, 68.1% of participants reported that training impacted menstrual function, including bleeding duration, bleeding volume, and menstrual cessation. This high incidence highlights an important issue for those working with this athlete population, as menstrual dysfunction may negatively impact normal skeletal development and other factors that increase current and future injury risk [31].

### Body composition, BMD, metabolic and reproductive hormone concentrations

This is the first study to identify LEA risk, and evaluate relevant indicators, for Chinese elite athletes. In the current investigation, for a sub-group of 14 elite athletes, the prevalence of EA, and associated factors were investigated, using a validated screening tool (LEAF-Q), and the objective quantification of biomarkers (metabolic hormones, BMD and body composition), which are currently recognized as some of the most valid and reliable methods of investigating EA in athletes [8, 11]. The results of our analysis indicated that for athletes at a high risk of LEA, E2 level was significantly lower, and the incidence of the primary amenorrhea and secondary amenorrhea were higher, when compared with athletes at low risk of LEA. Current evidence suggests that LEA causes disruption of Gonadotropin Releasing Hormone (GnRH) pulsation in the hypothalamus. This further alters luteinizing hormone (LH) and follicle-stimulating hormone (FSH), resulting in a decreased estradiol and progesterone concentration, ultimately resulting functional hypothalamic amenorrhea (FHA) [32]. Potentially, the assessment of biomarkers as used in the current investigation may provide some evidence for a more effective preventive screening procedure for LEA, which could contribute to the early detection of the health and performance sequela of LEA.

Results of the current investigation indicated that 42.9% of the Chinese elite athletes surveyed experienced an injury in the past year, the majority being muscle injury, joint injury and/or ligament sprain; only one athlete reported stress injury. The BMD data indicated that no participants in the current investigation had symptoms of low bone density (bone mineral density Z-score between − 1.0 and − 2.0) [4]. Compared to previous studies, the elite athletes in the current investigation had a lower incidence of low BMD [33, 34]. However, while BMD values were considered to be in the normal range for these elite athletes, the total-body BMD and BMD Z-score in the high-risk group were significantly lower than those in the low-risk group. It has been established that athletes’ BMD can influence long-term EA and menstrual status [35], and in the current investigation BMD was lower in LEA, although there was no significant correlation with E2. It has been established that LEA and menstrual disturbance can gradually impact bone health, leading to clinical endpoints such as osteoporosis and fractures [35]. We therefore recommend that future investigations focus on bone health in athletes, and specifically its association with injury, LEA and menstrual disorders.

## Limitations

Within this cross-sectional investigation, use of self-reported measures may have introduced bias, since this method is largely dependent upon on participants’ enthusiasm, compliance and memory [21]. Further, some sensitive questions may affect the authenticity of the response, the possibility of which may underestimate the risk of LEA. In this study, it was possible to collect data on biomarkers in a sub-group of only 14 elite athletes, in a limited number of sports. This relatively small sample size is one of the limitations of our study, and reflects the difficulty and challenge of recruiting national and world-class athletes, particularly those preparing for major international championship events. Thus, similar studies conducted in this area could be conducted with other athlete populations, and should include larger sample sizes, particularly for biomarker testing.

## Conclusions

Our study demonstrates that for Chinese female athletes, elite athletes have a higher risk of LEA than recreational athletes. Further, Chinese elite athletes, who have a higher training volume and training frequency than recreational athletes, have higher LEA risk, menstrual disturbance, and primary amenorrhea than recreational athletes. In addition, LEA risk was found to be associated with decreased E2 concentration and lower bone mineral density. These data may indicate that both elite and recreational groups may require additional education on nutritional strategies which can enhance both performance and health. In conclusion, emphasizing the importance of LEA, and promoting screening and prevention strategies is crucial to achieving the important goal of protecting the health of Chinese athletes involved in aesthetic sports, particularly given the association of LEA with and the potential for a negative impact on health.

## Data Availability

The datasets generated and/or analyzed as part of the current study are not publicly available due to confidentiality agreements with subjects. However, they can be made available solely for the purpose of review and not for the purpose of publication from the corresponding author upon reasonable request.

## Abbreviations

BMD: Bone mineral density
BMI: Body mass index
E2: Estradiol
ED: Eating disorders
EDI-3 RF: Eating Disorder Inventory-3 Referral Form
ELA: Elite athletes
FSH: Follicle-stimulating hormone
GnRH: Gonadotropin Releasing Hormone
LEA: Low energy availability
LEAF-Q: Low Energy Availability in Females Questionnaire
LH: Luteinizing hormone
REA: Recreational athletes
RED-S: Relative Energy Deficiency in Sport
T3: triiodothyronine
